# Improved total variation minimization method for few-view computed tomography image reconstruction

**DOI:** 10.1186/1475-925X-13-70

**Published:** 2014-06-05

**Authors:** Zhanli Hu, Hairong Zheng

**Affiliations:** 1 Paul Lauterbur Center for Biomedical Imaging Research, Institute of Biomedical and Health Engineering, Shenzhen Institutes of Advanced Technology, Chinese Academy of Sciences, Shenzhen, China; 2 Beijing Center for Mathematics and Information Interdisciplinary Sciences, Beijing, China; 3 Shenzhen Key Lab for Molecular Imaging, Shenzhen, China

**Keywords:** Computed tomography, Few-view, Adaptive prior image, Total variation, Compressive sampling

## Abstract

**Background:**

Due to the harmful radiation dose effects for patients, minimizing the x-ray exposure risk has been an area of active research in medical computed tomography (CT) imaging. In CT, reducing the number of projection views is an effective means for reducing dose. The use of fewer projection views can also lead to a reduced imaging time and minimizing potential motion artifacts. However, conventional CT image reconstruction methods will appears prominent streak artifacts for few-view data. Inspired by the compressive sampling (CS) theory, iterative CT reconstruction algorithms have been developed and generated impressive results.

**Method:**

In this paper, we propose a few-view adaptive prior image total variation (API-TV) algorithm for CT image reconstruction. The prior image reconstructed by a conventional analytic algorithm such as filtered backprojection (FBP) algorithm from densely angular-sampled projections.

**Results:**

To validate and evaluate the performance of the proposed algorithm, we carried out quantitative evaluation studies in computer simulation and physical experiment.

**Conclusion:**

The results show that the API-TV algorithm can yield images with quality comparable to that obtained with existing algorithms.

## Background

In recent years, the tremendous advances in computed tomography (CT) technology and applications have increased the clinical utilization of CT, creating concerns about individual and population doses of ionizing radiation. Studies indicate that dose from CT scans may have a lifetime attributable risk of cancer higher than previously assumed [1-3]. Reducing CT dose has been an area of active research in medical imaging [4]. Few-view CT image reconstruction is of great importance in clinical imaging for its potential to reduce the x-ray radiation dose to the human subject and the scan time. In few-view CT, less number of projection data than is required to satisfy the Nyquist sampling theorem is used. However, conventional filtered backprojection (FBP) based image reconstruction algorithms will occurs severe streak artifacts. Therefore, it is important to develop new algorithms in order to obtain more accurate images from few-view projections.

In 2006, a new image reconstruction theory, compressive sampling (CS), was proposed by Candes *et al.*[5-8]. They proved that an image of sparse signals could be satisfactorily reconstructed from far less measurements than what is usually considered necessary. Based on the fact that medical images are usually edge-sparse, total variation (TV) minimization is often used for solving incomplete data problems in tomography. Inspired by this work, many iterative algorithms for few-view CT image reconstruction have been proposed and investigated [9-16]. Sidky *et al.*[9,10] developed an adaptive steepest descent projection onto convex sets (ASD-POCS) algorithm based on an optimization strategy that minimizes the TV of the estimated image subject to data condition and other constraints. The ASD-POCS algorithm interleaves ART iterations with gradient descent steps for the TV penalty, aiming at the solution of the constrained minimization problem.

However, ASD-POCS algorithm does not directly incorporate a prior image into the reconstruction. In many applications of CT, prior CT projection data of the scanned object may be available. For example, in daily CBCT examinations for accurate patient setup and target localization in image-guided radiation therapy (IGRT), repeated scans have become routine procedures for the following days.

In this work, we develop an adaptive prior image TV (API-TV) algorithm for few-view CT image reconstruction. Similar to ASD-POCS, this algorithm is based on an interlaced iteration and is a simple combination of prior image. The API-TV algorithm follows the framework of ASD-POCS and has its own benefits. The paper is organized as follows. In section II, we detail describe the proposed API-TV algorithm. In Section III, we present results of our numerical studies by using both computer-simulation data and experimental data for validation and evaluation of the API-TV algorithm, respectively. The conclusion in Section IV.

## Methods

For CT image reconstruction from few-view data, the traditional filtered back-projection (FBP) method oftens suffers from serious streaking artifacts due to the ill condition of the number of projections. Recently, compressive sampling (CS) theory has been used to the CT reconstruction problem. In particular, total variation (TV) methods have demonstrated its power in image reconstruction from few-view projection. Sidky *et al.* presented an adaptive steepest descent projection on orthogonal convex subsets (ASD-POCS) algorithm for few-view reconstruction problem by solving the following constrained optimization problem

(1) $$f*=\arg\min{∥f∥}_{\mathrm{TV}}\mathrm{such}\mathrm{that}\left|\mathrm{Mf}-\tilde{g}\right|\leq \epsilon ,f\geq 0$$

where $\tilde{g}$ represents the projection data, *f* is the discrete image, *M* is the transfer matrix, and *ϵ* is the error tolerance parameter. The TV of the to-be-reconstructed image, i.e. ∥ *f* ∥_*TV*_ , is defined as:

(2) $${∥f∥}_{\mathrm{TV}}=\sum_{s,t,v}({\left(f_{s,t,v}-f_{s-1,t,v}\right)}^{2}+{\left(f_{s,t,v}-f_{s,t-1,v}\right)}^{2}+{\left(f_{s,t,v}-f_{s,t,v-1}\right)}^{2}){}^{1/2}$$

where *s*, *t* and *v* are the indices of the location of the discrete image.

Accordingly, the derivative of ∥ *f* ∥_*TV*_ is:

(3) $$\begin{array}{ll} \nabla_{f}{∥f∥}_{\mathrm{TV}}= & \frac{\partial {∥f∥}_{\mathrm{TV}}}{\partial f_{s,t,v}}\approx \frac{\left(f_{s,t,v}‒f_{s-1,t,v}\right)+\left(f_{s,t,v}‒f_{s,t-1,v}\right)+\left(f_{s,t,v}‒f_{s,t,v-1}\right)}{\sqrt{{\left(f_{s,t,v}‒f_{s-1,t,v}\right)}^{2}+{\left(f_{s,t,v}‒f_{s,t-1,v}\right)}^{2}+{\left(f_{s,t,v}‒f_{s,t,v-1}\right)}^{2}+\tau }}\\ & -\frac{\left(f_{s+1,t,v}‒f_{s,t,v}\right)}{\sqrt{{\left(f_{s+1,t,v}‒f_{s,t,v}\right)}^{2}+{\left(f_{s+1,t,v}‒f_{s+1,t-1,v}\right)}^{2}+{\left(f_{s+1,t,v}‒f_{s+1,t,v-1}\right)}^{2}+\tau }}\\ & -\frac{\left(f_{s,t+1,v}‒f_{s,t,v}\right)}{\sqrt{{\left(f_{s,t+1,v}‒f_{s-1,t+1,v}\right)}^{2}+{\left(f_{s,t+1,v}‒f_{s,t,v}\right)}^{2}+{\left(f_{s,t+1,v}‒f_{s,t+1,v-1}\right)}^{2}+\tau }}\\ & -\frac{\left(f_{s,t,v+1}‒f_{s,t,v}\right)}{\sqrt{{\left(f_{s,t,v+1}‒f_{s-1,t,v+1}\right)}^{2}+{\left(f_{s,t,v+1}‒f_{s,t-1,v+1}\right)}^{2}+{\left(f_{s,t,v+1}‒f_{s,t,v}\right)}^{2}+\tau }} \end{array}$$

Here *τ* is a relax parameter to keep the denominator not equal to zero.

In the proposed API-TV algorithm, the prior images can be first obtained from a prior scan. It could be an image reconstructed by a conventional analytic algorithm such as FBP algorithm from full-view projections. In summary, the pseudo-code for the presented API-TV algorithm is listed as follows:where the *α* is the control parameter and is selected to be 0.85. When *α* is set to be 0, the API-TV algorithm reduces to the conventional ASD-POCS algorithm. In line 1, an image obtained by a conventional FBP algorithm from full-view projections. In line 2, an initial estimate of the to-be-reconstructed image is set to be uniform with voxel value of 0. Each outer loop (lines 3–14) is performed by two separated iteration steps, i.e. the POCS (or the ART) (lines 4–10) and the gradient descent for the API-TV minimization (lines 11–13). After several general iterations, an accurate image reconstruction is obtained from few-view data samples.

1. Obtain prior image *f*_*p*_

2. Initial: $f_{s,t,v}^{\left(0\right)}=0$, *s*, *t*, *v* = 1, 2, …, *N*

3. repeat main loop

4. for ART iterations *i*=1:*I*

5.$f_{{}_{s,t,v}}^{\left(i\right)}=$ART_iteration ($f_{{}_{s,t,v}}^{\left(i-1\right)}$); *s*, *t*, *v* = 1, …, *N*

6. end ART

7. POCS: enforce positivity

8. if $f_{{}_{s,t,v}}^{\left(I\right)}>0$, $f_{{}_{s,t,v}}^{\left(I\right)}=f_{{}_{s,t,v}}^{\left(I\right)}$; *s*, *t*, *v* = 1, …, *N*

9. else $f_{{}_{s,t,v}}^{\left(I\right)}=0$; *s*, *t*, *v* = 1, …, *N*

10. end POCS

11. for TV gradient descent iterations *k*=1:*K*

12.

$$f_{{}_{s,t,v}}^{\left(k\right)}\approx \left(\alpha \cdot \nabla_{f}{∥f-f_{p}∥}_{{}_{\mathrm{TV}}}^{\left(k-1\right)}+\left(1-\alpha \right)\cdot \nabla_{f}{∥f∥}_{{}_{\mathrm{TV}}}^{\left(k-1\right)}\right)$$

13. end TV gradient descent

14. end if stop criterion is satisfy

To evaluate the differences between the results from ASD-POCS and API-TV, we performed computer simulations and experimental study in the following section.

## Results

### Simulation study

In this section, we validate our API-TV algorithm for cone-beam few-view reconstruction from noise-free and additive noisy projections. The high contrast Shepp-Logan phantom of an array size of 256 by 256 was used as the true image. The simulated data were generated from Shepp-Logan phantom with circular cone-beam scan trajectory. The radius of the circular trajectory was 500 mm and the source-to-detector distance was 1000 mm. The simulated detector had a flat-panel geometry of 512 × 512 with detection elements of size 1 × 1 mm^2^. A total of 60 projection views were simulated evenly over 360 degrees.

**Figure 1:**
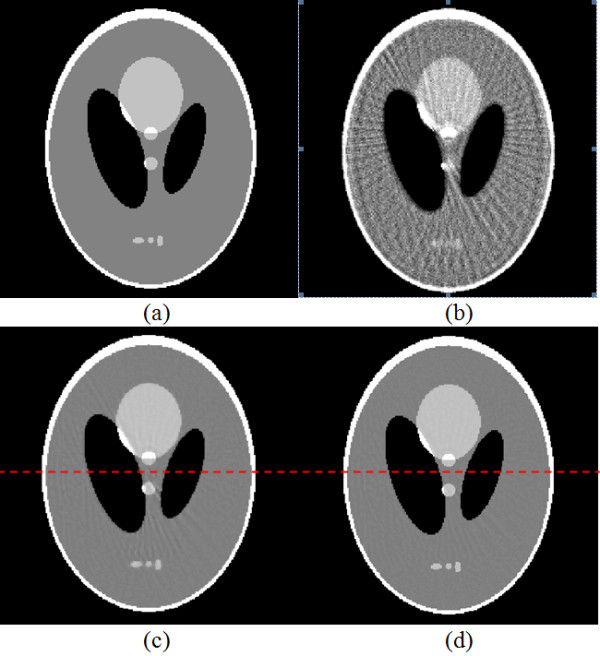
**A comparison study on the reconstructed images by conventional ART, ASD-POCS and API-TV algorithms respectively, each of which took 60 noise-free cone-beam projections and 50 iterations: (a) the true phantom image, (b) the reconstructed image by the conventional ART algorithm, (c) the reconstructed image by the ASD-POCS algorithm, and (d) the reconstructed image by the proposed API-TV algorithm.** The grey scale display range is [0.8, 1.2].

**Figure 2:**
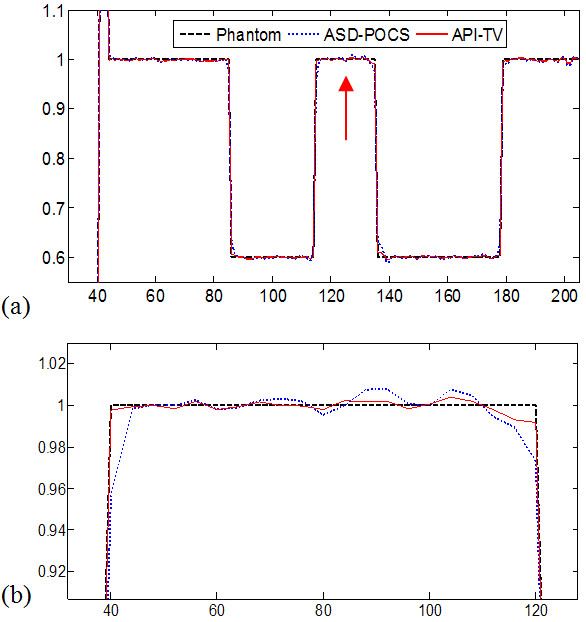
**The (a) shows the profiles of the reconstructed image from noise-free data crossing the 128th row and the (b) shows the zoomed images.** The true phantom (black dashed line), ASD-POCS (blue dot line), API-TV (red solid line).

A. *Noise-free cases*The images were reconstructed from 60 projection views selected. Figure 1 shows a comparison study of the reconstructed images by conventional ART, ASD-POCS and API-TV algorithms in the noise-free cases. It can be observed that the images reconstructed by the ASD-POCS and API-TV are visually much better than the results of conventional ART. The difference between the images from the ASD-POCS and API-TV algorithms can be observed in Figure 1(c) and (d), respectively. It can be observed that the proposed algorithms can produce high quality images with much less streak artifacts than the ASD-POCS results.To further visualize the difference between the two approaches in the cases of 60 projection views, their horizontal profiles are given in Figure 2 for a further illustration. The horizontal profiles of the resulting images were drawn across the 128th row for each approach and are shown in Figure 2, where the corresponding profile from the true phantom image is given for reference. It can be seen that the API-TV algorithm can achieve better profiles matching with the ideal ones than the ASD-POCS algorithm.

**Figure 3:**
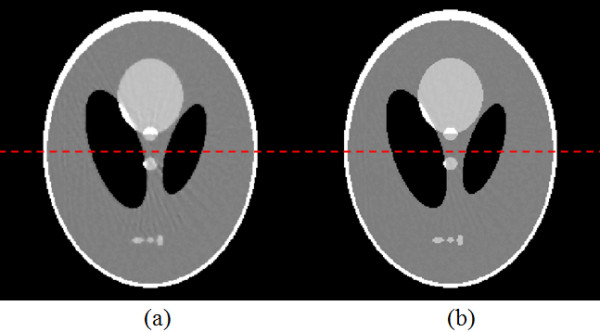
**A comparison study on the reconstructed images by ASD-POCS and API-TV algorithms respectively, each of which took 60 additive noisy cone-beam projections (about 10% white noise) and 50 iterations: (a) the reconstructed image by the ASD-POCS algorithm, and (b) the reconstructed image by the proposed API-TV algorithm.** The grey scale display range is [0.8, 1.2].

**Figure 4:**
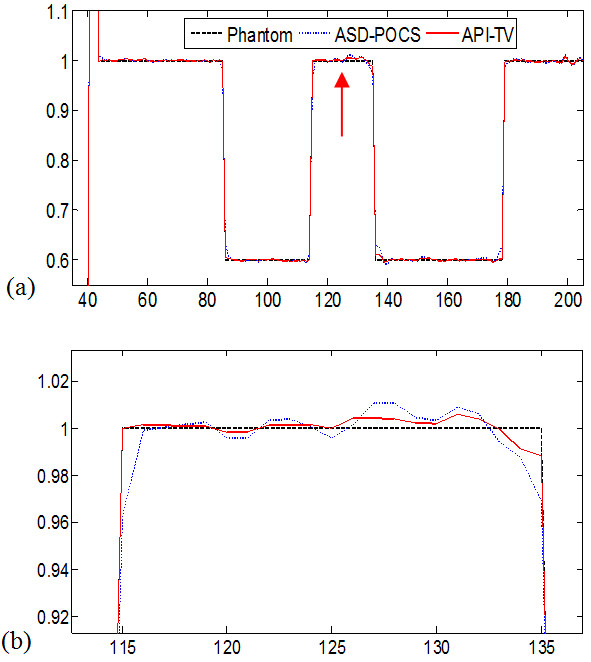
**Numerical simulation results with additive noisy projection data (about 10% white noise).** The **(a)** shows the profiles of the reconstructed image from noise data crossing the 128th row and the **(b)** shows the zoomed images. The true phantom (black dashed line), ASD-POCS (blue dot line), API-TV (red solid line).

B. *Noisy cases*In this section, image reconstruction from noisy data (about 10% white noise) was performed to analyze the robustness to noise of the API-TV algorithm. The parameters are the same as the noise-free situation. Figure 3 shows that the ASD-POCS images have more artifacts as compared to the images reconstructed by the API-TV algorithm from 60 projection views of the noisy data. Compared to the ASD-POCS algorithm, the API-TV algorithm preserved more edge details.Numerical simulation results with additive noisy projection data (about 10% white noise) are shown in Figure 4. The horizontal profiles of the images reconstructed in the cases of ASD-POCS and API-TV algorithms of noisy data along the 128th row are shown, respectively, with the corresponding profile of the true phantom image as a reference. These profiles also show that the API-TV algorithm preserved the edge details better than the ASD-POCS algorithm in the noisy cases for few-view reconstruction. These noisy simulation studies were consistent with our previous observations in the noise-free cases, and further concurred with the advantage of using the adaptive prior image for edge preservation in the API-TV model as compared to the conventional TV model. The results show the robustness of our algorithm for inconsistent data due to the presence of noise.

C. *Quantification based evaluation*

**Figure 5:**
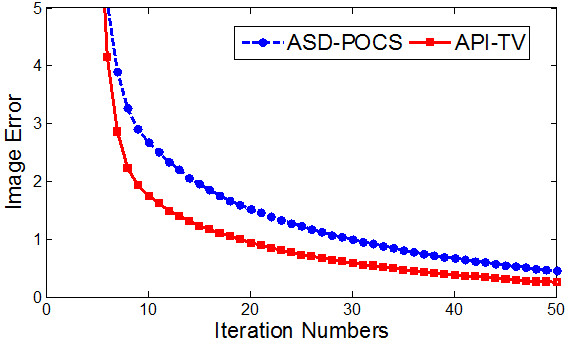
Plots of the image errors as a function of the number of iterations for the two algorithms respectively.

In addition to visualization based evaluation, we performed quantitative measure of the image error to evaluate the image quality. The convergence rates of the two algorithms were compared with each other, as quantified by the image error:

(4) $$E=\sqrt{\sum_{i,j,k}{\left(f_{i,j,k}^{*}-f_{i,j,k}\right)}^{2}}$$

where *E* denoted the image error, *f* is the reconstructed image, and f^*^ is the true image.As shown in Figure 5, the API-TV algorithm became converged after about 30 iterations, whereas the ASD-POCS algorithm took more iteration to converge. After 30 iterations, the image error was reduced to 0.592 using the API-TV method, much lower than 0.996 for the ASD-POCS method.

### Experimental study

A. *Prototype microCT system*

The developed MicroCT prototype system consisted of an x-ray source, a rotational object holder and a CCD detector. The open x-ray source (FXE: 160.51, YXLON, Germany) has a minimum focal spot size less than 2 μm and the focal spot size can been adjusted by changing the focusing current. A cooled x-ray CCD imaging detector (Quad-RO: 4320, Princeton Instruments, USA) was used to acquire the images. The CCD imaging detector has a high spatial resolution, large active imaging area and low noise (24 μm pixel size, 2084 × 2084 array and 50 × 50 mm^2^ active area).

B. *Small animal Imaging*

In order to validate the proposed algorithm’s performance, we used the laboratory mouse to obtain projection data. Circular cone-beam data were acquired from the mouse of 120 projection views distributed over 2*π*, with the parameters of 80 kVp, 7w, and 8 s per exposure. The source-to-detector distance (SDD) was held at 710 mm and the source-to-object distance (SOD) was set at 530 mm.

**Figure 6:**
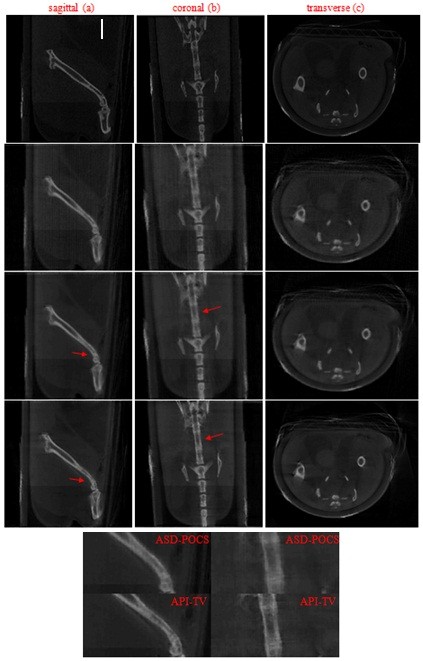
**Images of the laboratory mouse within sagittal (a), coronal (b), and transverse (c) slices reconstructed from the 120-view data by use of the (row 2) ART, (row 3) ASD-POCS, and (row 4) API-TV algorithms, respectively.** The FBP method using 360 views of projections as full-view prior image (row 1). The zoomed ROIs indicated by the red arrows in (row 3) and (row 4) are displayed at the bottom. The grey scale display range is [0.0, 0.04].

A full-view prior image was reconstructed using the FBP algorithm. The full-view scan was designed to take projection data at 360 views on regular interval. The few-view scan was designed to take projection data set at 120 views on regular interval. The few-view scan implies that dose delivered to the scanned object using few scan is roughly 1/3 times of the full scan.In Figure 6, we display images reconstructed from the 120-view data by use of the ART, ASD-POCS, and API-TV algorithms, within transverse (z = 0 mm), coronal (x = 5.088 mm), and sagittal (y = 5.188 mm) slices. Visual inspection of reconstructions in Figures 6 suggests that the ASD-POCS and API-TV algorithms can effectively suppress streak artifacts and noise observed in images obtained with the ART algorithms. In addition, the result of the API-TV shows more details on the edges than the result of the ASD-POCS as indicated by the arrows in figures.

## Conclusion

In this paper, we introduced a novel adaptive prior image total variation (API-TV) minimization model for low-dose CT image reconstruction from few-view projection measurements. We have evaluated and demonstrated the performance of our algorithm in a number of few-view reconstruction problems. Numerical studies on both the computer simulation and micro-CT imaging experiments were taken to validate our algorithm. In all the situations shown, API-TV algorithm leads to significant artifact reduction without visible over smoothing of low contrast areas in the image. All results were compared to a previously proposed implementation of the ASD-POCS algorithm.

A weakness in our proposed API-TV algorithm is that it assumes that the patients were at exactly the same position during the repeated scans. This assumption, however, may not always be the case as the patient is frequently repositioned for optimal imaging in image-guided radiation therapy (IGRT) applications. It is therefore unavoidable that mismatched areas between current and prior images may occur in practice. Therefore, a robust implementation of this algorithm will require accurate registration and voxel consistency for each projection.

In the present study, because of the limitations of the experimental conditions, many clinical conditions were not considered. Future work is therefore needed to verify proposed algorithm on real clinical projections. We hope that the present API-TV algorithm may be widely used in medical clinic.

## Competing interests

The authors declare that they have no competing interests.

## Authors’ contributions

ZH worked on the algorithm design and implementation, and wrote the paper. HZ contributed to discussion and suggestions throughout this topic. Both authors read and approved the final manuscript.
